# Distal Humerus Metaphyseal-Diaphyseal Junctional Fracture of a Four-Year-Old Child Treated With Intramedullary Steinmann Wire Fixation: A Case Report

**DOI:** 10.7759/cureus.47949

**Published:** 2023-10-30

**Authors:** Muhammed Yusuf Afacan, Abdisalam Mutaj Shafaj Nur, Tural Vahitoglu

**Affiliations:** 1 Department of Orthopaedics and Traumatology, Istanbul University-Cerrahpasa, Cerrahpasa Faculty of Medicine, Istanbul, TUR; 2 Department of Orthopaedics and Traumatology, Silvan Dr. Yusuf Azizoglu State Hospital, Diyarbakir, TUR

**Keywords:** pediatric distal humerus fracture, pediatric elbow fracture, childhood diaphyseal humerus fracture, metaphyseal-diaphyseal junctional humerus fracture, open lateral approach, intramedullary steinmann wire fixation

## Abstract

Distal humeral metaphyseal-diaphyseal fractures are rare and inherently unstable injuries. Non-operative treatments can make it hard to maintain reduction. Open or closed reduction with percutaneous K-wire fixation may be the preferred treatment option for these fracture types. This case report describes successfully managing a rare distal humerus metaphyseal-diaphyseal junctional (MDJ) fracture in a four-year-old child using intramedullary Steinmann wire fixation. A four-year-old male child applied to the emergency service with a swollen elbow. He had a history of trauma 10 days ago. There was a long arm splint on his arm. A displaced distal MDJ fracture of the left humerus was detected on the radiograph. Due to its instability, we preferred surgical management. With a lateral incision, we obtained a successful reduction after manipulation. Subsequently, we achieved the anatomical reduction with three Steinmann pins. We applied two Steinmann pins intramedullary, and the other one crosses from the medial epicondyle and exits the lateral cortex, forming a crossed-pin configuration at the fracture site. We immobilized the extremity for four weeks with a long arm splint. At the end of the fourth week, we removed the Steinmann pins. After removing the wires, we began an active range of motion exercises. The plain X-ray at the two-month follow-up revealed good fracture healing with no residual elbow deformity. The patient could perform a complete elbow range of motion. The case highlights the challenges in treating pediatric distal metaphyseal-diaphyseal humerus fractures, and it demonstrates the effectiveness of this intramedullary Steinmann wire fixation technique in achieving stable fracture reduction and promoting rapid healing in a small child.

## Introduction

The most frequent elbow fracture in children is the supracondylar humeral fracture, which is 55-75% of all pediatric elbow fractures [1,2]. The standardized treatment protocol for supracondylar humerus fracture is non-surgical immobilization for the non-displaced and closed reduction and percutaneous pinning for the displaced fractures [3,4]. However, when the fracture line crosses above the olecranon fossa, the fracture is termed a distal metaphyseal-diaphyseal junctional (MDJ) fracture [5]. Fractures in the distal humeral diaphysis and MDJ fractures are considerably rarer when compared to fractures occurring in the supracondylar humeral region [6]. The treatment of diaphyseal fractures poses significant challenges due to the distal diaphysis’ triangular shape and thinner periosteum compared to the supracondylar area [7,8]. Managing these fractures without operation is complex and problematic. Therefore, open reduction was typically recommended as the preferred treatment approach. In this case, we present a four-year-old child who sustained a rare injury of distal MDJ humerus fracture. He was successfully managed using open reduction and intramedullary Steinmann wire fixation. We also aimed to show that Steinmann pins could be used as intramedullary fixation, resembling intramedullary nail fixation, and the results were successful.

## Case presentation

**Figure 1:**
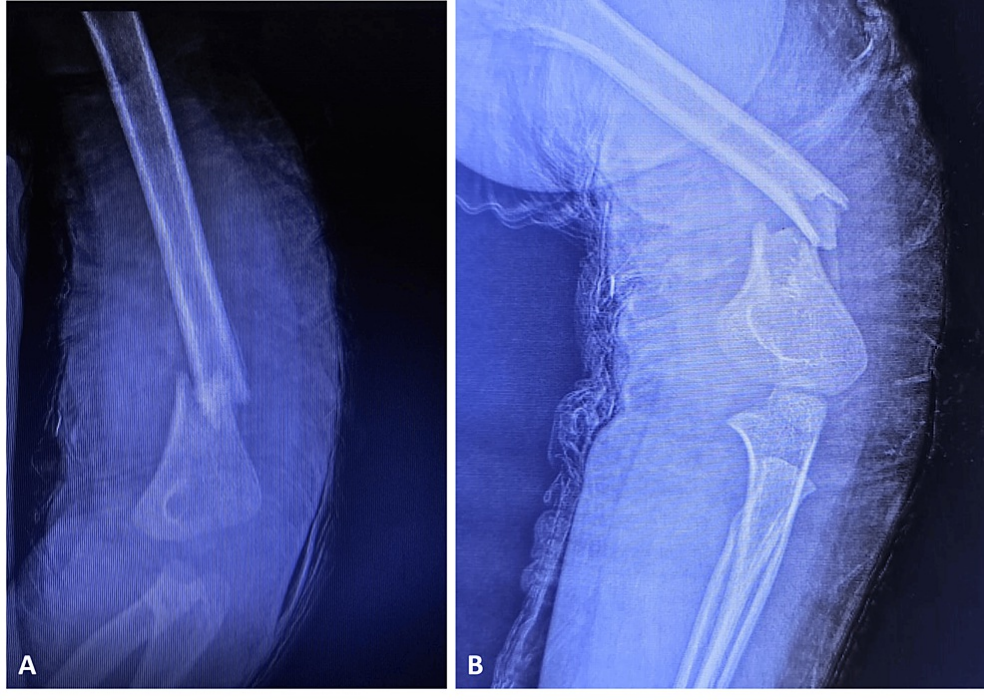
Elbow radiographs of the patient before the operation Elbow anteroposterior (Figure 1A) and lateral (Figure 1B) views of the patient before the operation showed a distal humerus metaphyseal-diaphyseal junctional displaced fracture.

We present a unique case of a four-year-old male child applying to the emergency department with a painful and quite swollen left arm. The patient had a history of a fall on his left elbow 10 days ago and was applied a long arm splint by the primary care physician. Physical examination revealed pain, tenderness, deformity, abnormal movement, ecchymosis, and swelling along the midshaft and distal part of the left humerus with an intact neurovascular status. The radiographic evaluation confirmed a displaced distal MDJ fracture of the left humerus (Figure 1). 

**Figure 2:**
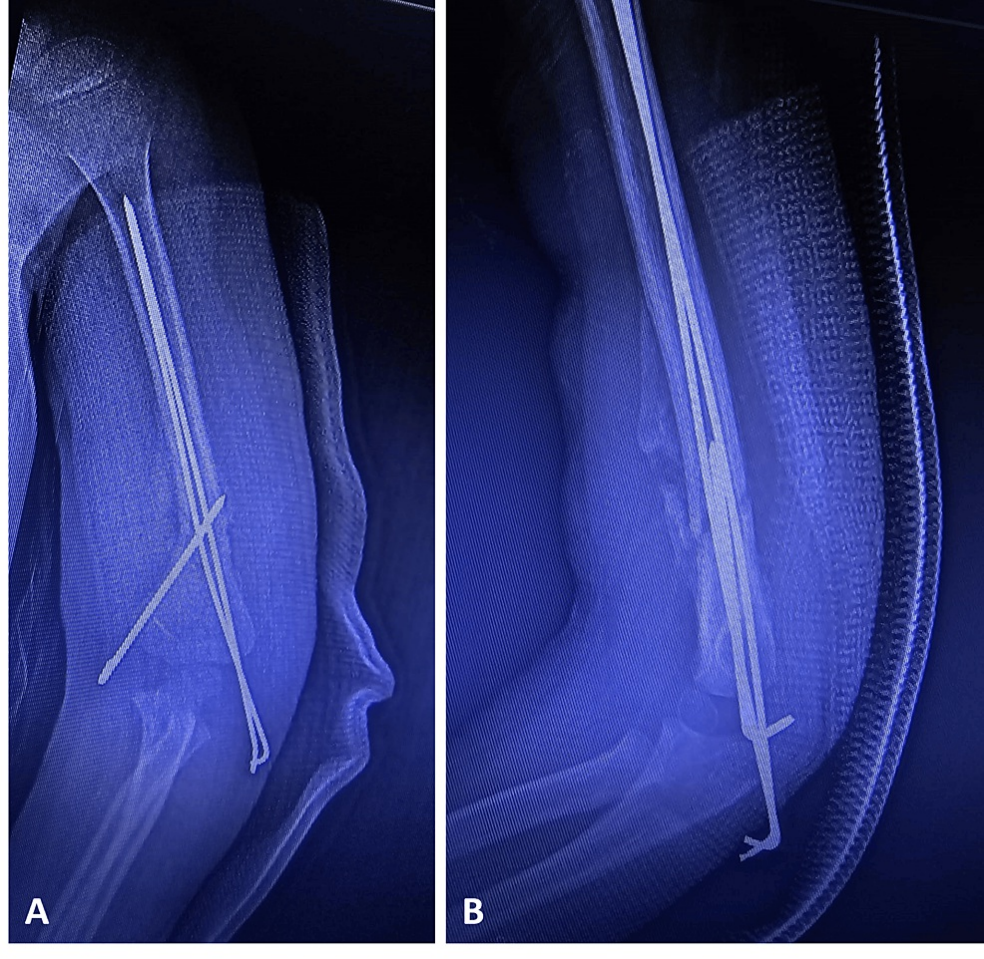
Elbow radiographs of the patient after the operation Elbow anteroposterior (Figure 2A) and lateral (Figure 2B) views of the patient after the operation, showing distal humerus metaphyseal-diaphyseal junctional fracture fixation with Steinmann pins with proper alignment.

Due to the inherent instability of the distal MDJ fracture of the humerus, we preferred surgery and performed open intramedullary fixation with Steinmann wires. Under general anesthesia, we first attempted a closed reduction, but it was unstable, and we could not maintain the reduction. We made a lateral incision to reach the fracture site. Once we obtained adequate reduction after manipulation, we performed intramedullary fixation using a 2 mm Steinmann pin. The wire was inserted axially into the distal fracture fragment under fluoroscopic guidance. Subsequently, the extremity was put under axial traction, during which we corrected the coronal alignment, overlap, and mediolateral translation using anteroposterior (AP) C-arm fluoroscopy. Once we rectified the frontal displacement, an assistant held the child’s elbow steady to maintain the reduction, ensuring no arm movement. Then, we rotated the C-arm by 90° to capture a lateral image of the elbow. We flexed the elbow to address sagittal angulation and translation and made anterior and posterior adjustments as necessary. During this stage, we exercised caution to minimize manipulation, closely monitoring the process via fluoroscopy. This caution was essential due to the vulnerability of the periosteum at the MDJ site since excessive manipulation could tear the periosteum, and excessive flexion of the injured elbow might compromise the periosteum’s integrity. Following this, we inserted the pre-pinning Steinmann wire across the fracture site and into the proximal marrow cavity while the assistant ensured that the elbow remained stable. After confirming the reduction under AP, oblique, and lateral fluoroscopy, we percutaneously inserted 2 mm Steinmann wire, crossing from the medial epicondyle, forming a crossed-pin configuration at the fracture site, and exiting the lateral cortex of the humerus. We performed elbow flexion, extension, and rotation to verify the stability of the fracture post-fixation (Figure 2). 

**Figure 3:**
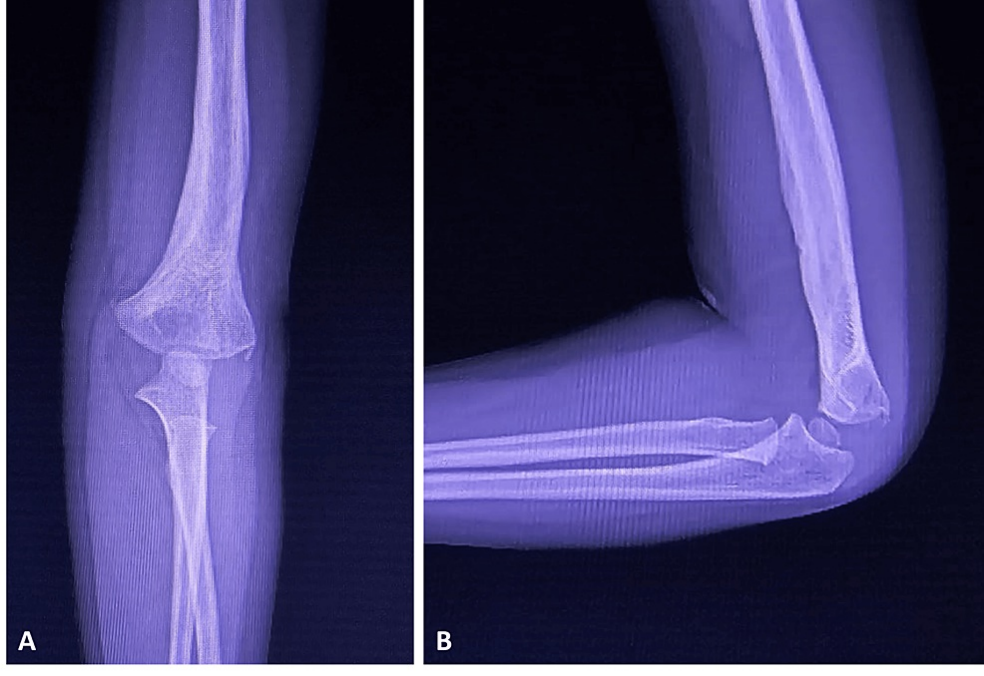
Elbow radiographs of the patient in the second month after the operation Elbow anteroposterior (Figure 3A) and lateral (Figure 3B) views of the patient in the second month after the operation, showing proper alignment and union.

**Figure 4:**
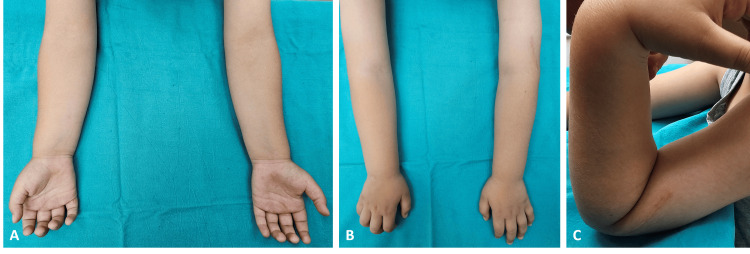
Elbow photographs of the patient in the second month after the operation Anterior elbow photograph of the patient in the supinating forearm (Figure 4A), in the pronating forearm (Figure 4B) with full elbow extension, and lateral elbow photograph of the patient in the flexing elbow (Figure 4C) in the second month after the operation.

Postoperatively, we used a long arm splint to immobilize the upper extremity for four weeks. Subsequently, we removed the long arm splint and the Steinmann pins, and the patient started on active range of motion exercises. At the second-month follow-up, the plain X-ray showed satisfactory healing with no residual elbow deformity (Figure 3) and full elbow range of motion (Figure 4), although we started the range of motion exercises in the fourth week. 

## Discussion

Management of distal humeral metaphyseal-diaphyseal fractures can be very challenging because the distal humerus diaphysis has a thinner periosteum, and the humerus diaphysis is more triangular compared to the supracondylar region [7]. The thin periosteum surrounding the MDJ does not provide a competent hinge for manual reduction. The proximal location of the MDJ fractures that results in an extended lever arm leads to the potential displacement of the fracture by minor forces [4]. From an anatomical perspective, the cross-sectional area of the bone at the MDJ site is considerably smaller compared to the supracondylar humerus region. Consequently, there is a reduced contact surface available for maintaining the reduction. Due to these anatomical and biomechanical characteristics of the MDJ, fractures in this region exhibit greater instability in contrast to supracondylar fractures [4]. Because humerus diaphyseal fractures are extremely unstable, reducing them is extremely difficult while maintaining alignment. Therefore, open reduction is recommended [9]. In addition, diaphyseal fractures tend to heal slowly, requiring a longer immobilization time, and are prone to postoperative complications [7].

The MDJ humerus fracture in our patient was fixed intramedullary with Steinmann pins to ensure proper alignment, increase stability, and prevent rotational deformity. Although closed reduction and percutaneous pinning were recommended as a new approach by Zhou et al. [4], we achieved reduction with a small lateral incision. In this way, we overcome the difficulties in fixing MDJ humerus fractures. Tomori et al. reported a six-year-old child with MDJ fracture, treated with open reduction and K-wire fixation. However, they used the anterior approach in open reduction [8]. This case resembled our case in many manners. However, we used a lateral approach instead, which was safe and easy to perform. They also immobilized the patient for four weeks, as in our case, and then started on the range of motion exercises. We did the same because we did not want to lose reduction by early movement. The child tolerated the long immobilization period well, and the range of motion was the same as the other side at the end of the sixth week. Elastic stable intramedullary nailing (ESIN) is also among the treatments of choice in MDJ humerus fractures, maintaining a stable reduction without rotation and ensuring early mobilization. Marengo et al. evaluated the surgical outcomes of ESIN in MDJ humerus fracture patients and ended up with successful results [10]. However, their patients' average age was 9.7 years. Since our patient was four, we preferred the Steinmann pins as intramedullary fixation rather than ESIN and removed the pins in the fourth week.

## Conclusions

We presented excellent results with a four-year-old child experiencing an MDJ distal humerus fracture. The open lateral approach may ease the reduction and stability preservation. The Steinmann pins took on the intramedullary nails duty for preserving the alignment, rotational stability, and reduction. The importance of this case is that the MDJ humerus fracture was observed at such a young age, and its surgical treatment was performed with a lateral approach and intramedullary Steinmann pins. The results are satisfactory with this easy-to-apply method, and we recommend further studies with this young age performing similar surgery to support the outcomes.
